# A Two-Step Growth Pathway for High Sb Incorporation in GaAsSb Nanowires in the Telecommunication Wavelength Range

**DOI:** 10.1038/s41598-017-09280-4

**Published:** 2017-08-31

**Authors:** Estiak Ahmad, Md Rezaul Karim, Shihab Bin Hafiz, C Lewis Reynolds, Yang Liu, Shanthi Iyer

**Affiliations:** 1 Joint School of Nanoscience and Nanoengineering, North Carolina A&T State University, Greensboro, NC 27401 USA; 2 Department of Electrical and Computer Engineering, North Carolina A&T State University, Greensboro, NC 27411 USA; 3 Department of Materials Science and Engineering, North Carolina State University, Raleigh, NC 27695 USA

## Abstract

Self-catalyzed growth of axial GaAs_1−x_Sb_x_ nanowire (NW) arrays with bandgap tuning corresponding to the telecommunication wavelength of 1.3 µm poses a challenge, as the growth mechanism for axial configuration is primarily thermodynamically driven by the vapor-liquid-solid growth process. A systematic study carried out on the effects of group V/III beam equivalent (BEP) ratios and substrate temperature (T_sub_) on the chemical composition in NWs and NW density revealed the efficacy of a two-step growth temperature sequence (initiating the growth at relatively higher T_sub_ = 620 °C and then continuing the growth at lower T_sub_) as a promising approach for obtaining high-density NWs at higher Sb compositions. The dependence of the Sb composition in the NWs on the growth parameters investigated has been explained by an analytical relationship between the effective vapor composition and NW composition using relevant kinetic parameters. A two-step growth approach along with a gradual variation in Ga-BEP for offsetting the consumption of the droplets has been explored to realize long NWs with homogeneous Sb composition up to 34 at.% and photoluminescence emission reaching 1.3 µm at room temperature.

## Introduction

The one-dimensional (1D) architecture of nanowires (NWs) enables high tolerance of lattice mismatched heterostructures, sub-wavelength optical phenomena, and quantum size effects^1–3^ When combined with an ability to integrate with other nanoscale and microscale devices, 1D NWs provide the ability to fabricate optoelectronic devices with distinct properties. Nanowires are commonly grown via the vapor-liquid-solid mechanism using gold (Au) as a catalyst. However, Au induces mid-gap levels in Si-compatible technological processes, which can be deleterious to device performance. Self-assisted (Au-free) growth overcomes these issues and yields NWs of high structural and optical qualities that are potentially scalable for mass production.

Among nanowires of different semiconductor material systems, the bandgap of GaAs_1−x_Sb_x_ covers the important wavelength range from 870 nm (GaAs) to 1700 nm (GaSb), which has potential applications in next generation optoelectronic devices, namely solar cells, optical telecommunications, photonic integrated circuits and quantum information science^4–8^. They also belong to the class of Sb-based III-V compound semiconductor NWs with favorable optoelectronic characteristics, namely high optical absorption and superior carrier mobility. GaAsSb NWs can be grown in both axial and core-shell architectures. One of the favorable attributes of GaAsSb NWs is that the NWs exhibit pure ZB crystal structure over the entire composition range^9^. Bandgap tuning corresponding to the desirable wavelength of 1.3 µm in the telecommunication window has been demonstrated^10, 11^ with a core-shell configuration. However, these nanowires suffer from a high density of planar defects with detrimental impact on the optoelectronic quality of the NWs. In contrast, axially configured GaAsSb NWs yield nearly planar-defect-free nanowires due to the predominance of the thermodynamically driven vapor-liquid-solid (VLS) growth mechanism, which leads to improved optoelectronic quality nanostructures^12^. Even though there is an extensive body of literature on Au-catalyzed and Au-free axial GaAsSb NWs^12–23^, the majority of the work on self-assisted axial GaAsSb NWs is mainly focused on structural and electronic characterization. Despite the fact that in the axial configuration an Sb composition as high as 93 at.% has been reported^9^, Sb compositions beyond 30 at.% have a detrimental impact on NW morphology and characteristics, namely uneven growth, tapered morphology, multiple facets, compositional gradient, thick parasitic islands of GaAsSb and poor PL emission^9, 12, 19–21^.

In this paper, we report on a novel pathway to incorporate higher atomic concentrations of Sb in GaAs_1−x_Sb_x_ axial NWs grown by self-assisted epitaxy without having any adverse impact on NW density and optical emission. A systematic study on the impact of different MBE growth parameters on Sb incorporation in the NWs is presented. These experimental studies, along with qualitative analysis using an analytical relation between effective vapor composition and NW chemical composition, not only enabled better understanding on the influence of different growth parameters on NW growth but also provided insight to a two-step growth temperature process with a corresponding Ga flux variation during growth. Although a two-step growth technique is not new for thin films^24–27^ and NW synthesis by chemical vapor deposition (CVD) technique^26–28^, it is not common for NWs grown by MBE^29^. It has been reported that both binary and ternary III-V NWs synthesized by two-step CVD method exhibited long, straight, limited radial growth and excellent crystallinity when compared to NWs grown at invariant growth temperature via a single-step CVD growth technique^28, 30, 31^. We present in this work a study of the two-step growth temperature approach in conjunction with Ga variation during the growth, using a variety of characterization techniques, to attain NWs of good compositional homogeneity with PL emission reaching 1.3 μm.

## Experimental Details

The growth of the NWs was carried out in an EPI 930 solid-source MBE system using As_4_ and Sb_2_ as the group V constituent sources. The self-catalyzed NWs were grown on chemically cleaned (Piranha/HF) p-type Si (111) substrates at 620 °C with a constant group V beam equivalent pressure (BEP) of 4.8 × 10^−6^ Torr and group III BEP of 2.4 × 10^−7^ Torr (unless otherwise mentioned)^11, 32, 33^. Growth was initiated by opening the Ga shutter 15 sec prior to opening of the As and Sb shutters. The V/III ratio was maintained constant at 20 for the reference sample corresponding to an Sb composition of 16 at.%. In all the growth activities, the Sb flux was held constant, while other growth parameters, namely substrate temperature, As and Ga flux, were varied. Growth was performed for 20 minutes and terminated by closing the Ga shutters, while keeping Sb and As shutters open until the substrate temperature was lowered to 570 °C in order to solidify the Ga droplets on the NW tips.

Scanning electron microscope (SEM) imaging was performed using a Carl Zeiss Auriga-BU FIB field emission scanning electron microscope (FESEM). The scanning transmission electron microscopy (STEM) – energy dispersive x-ray spectroscopy (EDS) analysis was performed on an aberration-corrected (probe) FEI Titan G2 system operated at 200 kV. The bright field TEM (BF-TEM), selected-area electron diffraction (SAED), and high-resolution TEM (HRTEM) were characterized on a JEOL 2010F microscope and a FEI Titan microscope, both operated at 200 kV. X-ray diffraction scans (XRD) were performed using a Bruker D8 Discover instrument with a DaVinci diffractometer in the standard Bragg-Brentano para-focusing configuration. The µ-photoluminescence (µ-PL) spectra of the NWs were measured by using a 633 nm He-Ne laser as the excitation source, with a 0.32 m double grating monochromator for wavelength dispersion, an InGaAs detector and conventional lock-in amplifier techniques. An Olympus IR 50× lens was used to focus the laser on the NWs. A closed-cycle optical cryostat from Montana Cryostation, with the sample chamber interfaced with a fiber-coupled confocal microscope, was used to determine the PL characteristics at 4 K. Raman spectroscopy was performed at room temperature in a Horiba Jobin Yvon ARAMIS Raman microscope with a He-Ne laser (633 nm) excitation source.

## Results and Discussions

The organization of this section is as follows: effects of V/III BEP ratio and substrate temperature variation on NW density and composition, discussion of these results using analytical formulation of constituents and vapor composition, followed by systematic study of the two-step growth process using a variety of characterization techniques.

### V/III BEP Ratio

**Figure 1:**
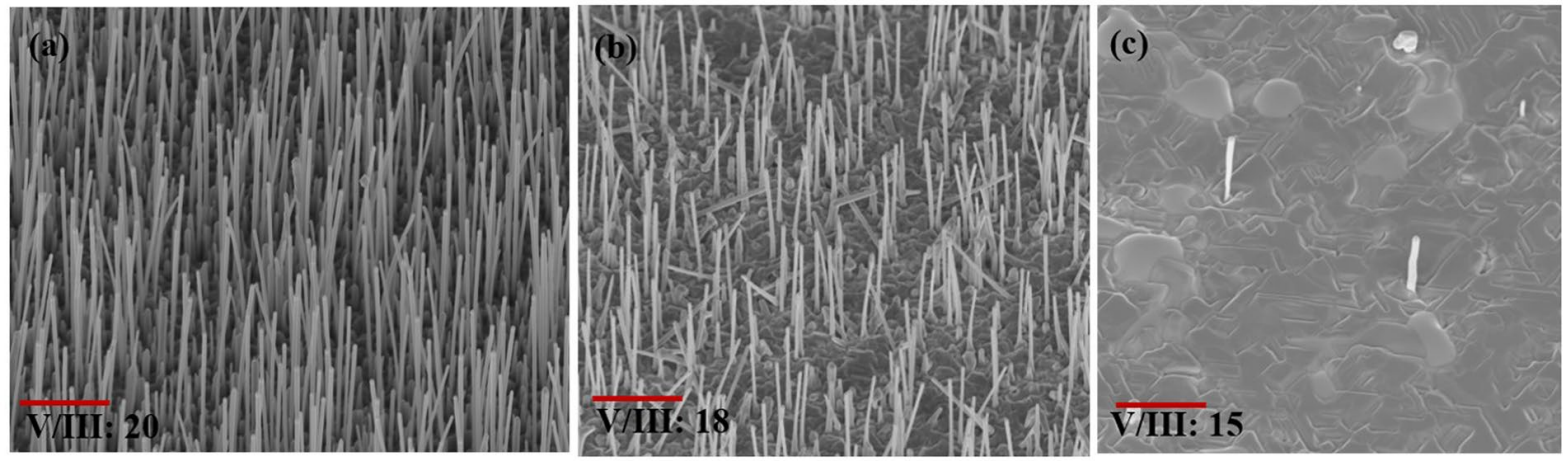
(**a**), (**b**) and (**c**) SEM images of GaAsSb with V/III ratio 20, 18 and 15, respectively (scale bar 2 µm).

Growths were carried out at 620 °C for V/III ratios of 20, 18 and 15 by changing only the As flux with the Sb and Ga fluxes remaining invariant (see Fig. 1). For the V/III ratio decrease from 20 to 18, the axial growth rate increased from 2.1 nm/s to 2.5 nm/s with a consequent reduction in NW density from 4 × 10^8^ cm^−2^ to 1 × 10^8^ cm^−2^, respectively. With further lowering of the V/III BEP ratio to 15, a drastic reduction in the NW density (5 × 10^6^ cm^−2^) at the expense of 2D growth and the presence of excess Ga were observed (see Fig. 1c). We attribute the observed reduction in NW density to a lowering of the chemical potential (Δµ) with a decrease in the V/III BEP ratios. For a dilute solution of group-V atoms in the Ga droplet, the chemical potential can be expressed as ${\rm{\Delta }}\mu =\,\mathrm{ln}(c/{c}_{e})$ ^34^. Here, *c* represents the concentration of the group-V atoms in the droplet and *c*_*e*_ is the value of *c* for the alloy at equilibrium. It should be noted that the low solubility of As atoms which is in excess^35^ justifies the assumption of the dilute solution here. The dependency of the nucleation rate (F_n_) of the metastable complex of group-III and group-V atoms on chemical potential can be described by the following relationship^36^:

1 $${F}_{n}\propto exp[-\frac{1}{{\rm{\Delta }}\mu }]$$

Equation 1 is a reasonable approximation of the more complex actual expression. Here, the change in chemical potential caused by variation in V/III BEP ratio is assumed to be the dominant term influencing the 2D nucleation rate^36^. Lowering the V/III BEP ratio would reduce the amount of group-V species collected by the droplet which in turn would reduce the combined atomic concentration *c* of the group-V species in the droplet and consequently a greater number of droplets fail to nucleate due to the concomitant lowering in chemical potential. Eventually, these NWs get buried under the laterally grown layer on the substrate which results in severe reduction in the NW density.

The PL peak energies for the V/III ratios of 20 and 18 did not reveal any significant shift in the 4 K and room-temperature PL peak values, which were observed at 1.11 eV and 1.09 eV, respectively (not shown here; see ref. 22). The Sb concentration determined from EDS was ~16 at.% for both of these samples.

### Substrate Temperature

**Figure 2:**
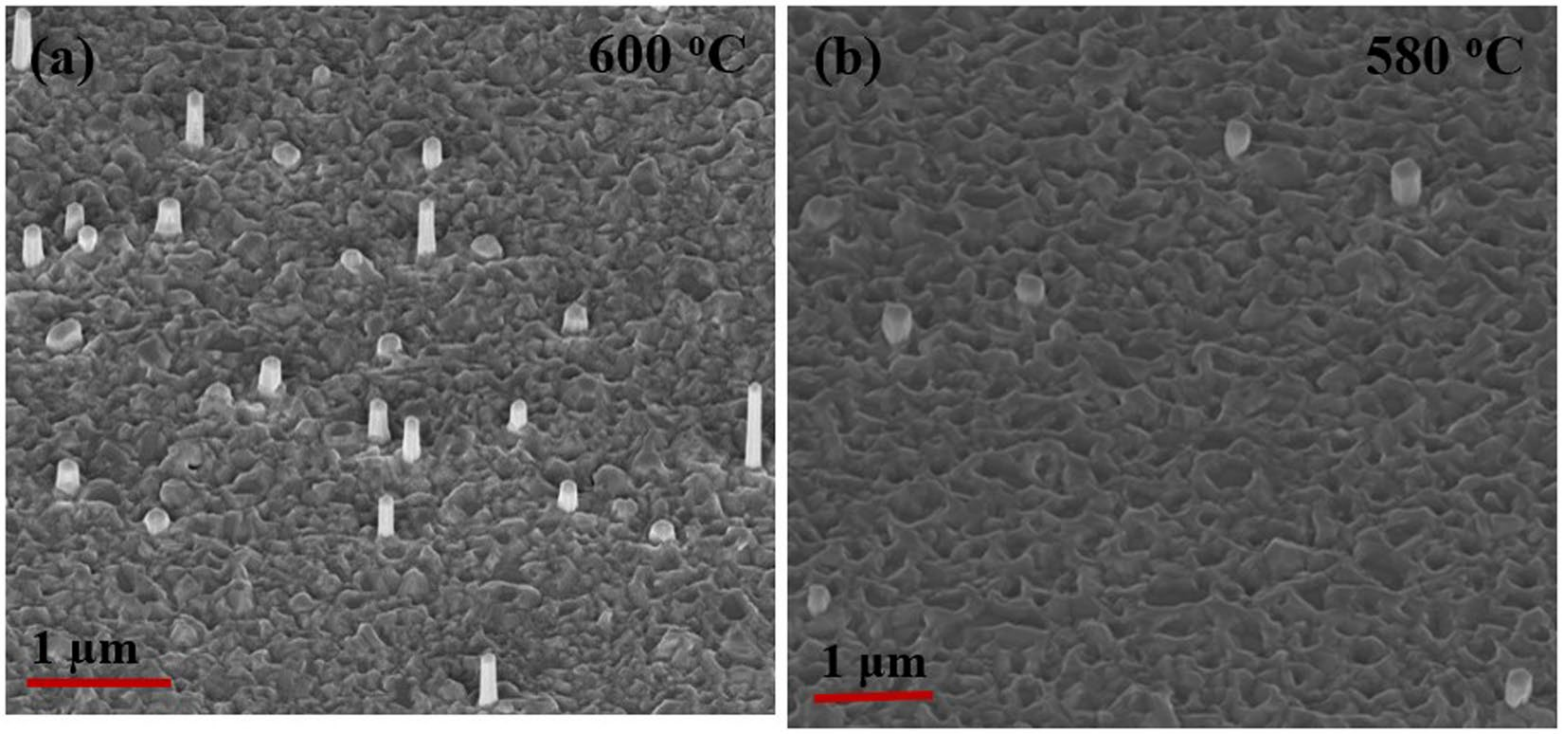
(**a**) and (**b**) show the GaAsSb NWs grown at substrate temperatures of 600 °C and 580 °C, respectively.

**Figure 3:**
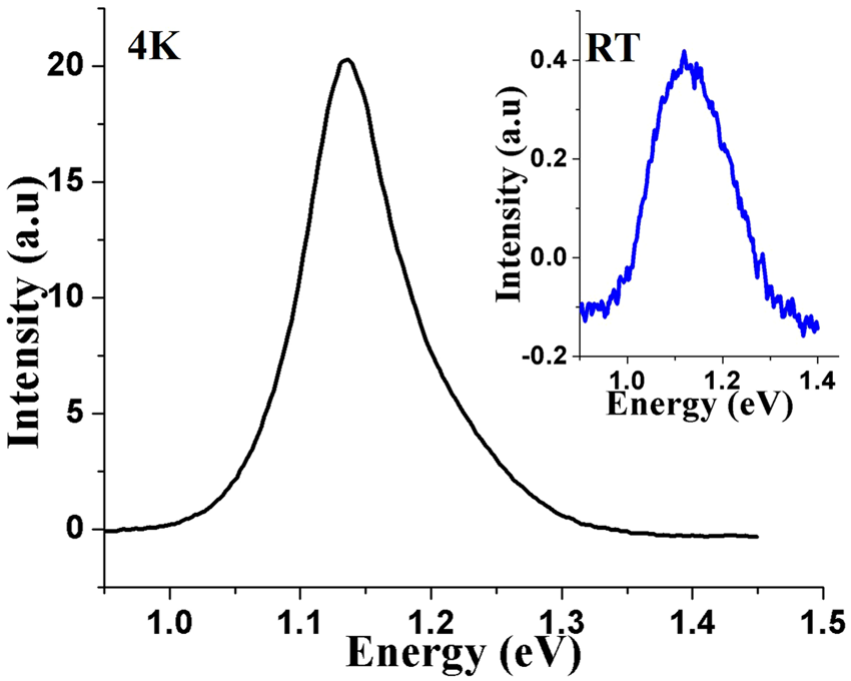
Photoluminescence spectra of GaAsSb NWs with Sb composition of 16 at.% (reference sample); inset shows the corresponding room-temperature PL spectra.

Using the optimized V/III ratio of 20 for maximum nanowire density, the effects of substrate temperature reduction from 620 °C to 600 °C and then 580 °C were examined. Figure 2 shows the corresponding SEM images of GaAsSb axial NWs at the two lowest temperatures. A significant reduction in the density of NWs (5 × 10^7^ cm^−2^ and 1 × 10^7^ cm^−2^ for 600 °C and 580 °C, respectively) was observed with a concomitant change in the NW physical dimensions with a decrease in substrate temperature. The substrate temperature affects NW density in a number of ways. First, an increase in the number density of the Ga droplet with a decrease in temperature^37^ is expected from reduced mobility of Ga adatoms. However, it also causes the Ga droplet volume to be smaller^37^, which can alter the contact angle between the droplet and the substrate surface^38^ as well as the amount of growth species intercepted by the droplet^23^. The reduction of the NW density with decrease in growth temperature therefore suggests that the reduced desorption of the Ga adatoms from the substrate may balance the reduction in droplet volume to some extent, but it cannot compensate for other effects. These have a deleterious impact on nanowire nucleation^39^. Therefore, we suggest that lowering the substrate temperature to 600 °C results in a reduction in the density of the droplets, which can retain the optimum volume for NW nucleation, leading to uneven 2D growth. Note that new Ga droplets can form during growth^37^ on the substrate surface roughened by lateral growth, which is likely responsible for the variation in NW lengths (see Fig. 2a). A further decrease in temperature to 580 °C results in complete 2D growth. The lengths and diameters of the NWs were observed to be ~2.5 µm and ~120 nm, 1.7 µm and 150 nm, and 0.5 µm and 190 nm, respectively for substrate temperatures of 620 °C, 600 °C and 580 °C, respectively. The corresponding Sb concentration varied from 16 at.%, 26 at.% to 30 at.%. The 4 K photoluminescence (PL) spectra were measured (Fig. 3) only for 16 at.% Sb; Sb incorporation was as expected with peak emission occurring at 1.13 eV while the room-temperature data exhibited a 20 meV red shift with respect to that at 4 K.

The increase in the Sb atomic percentage in the NWs with the decrease in substrate temperature can be explained using the following equation (see the supplementary information for derivation of the equation):

2 $$\frac{1}{{\boldsymbol{x}}}=\chi (\frac{1}{\varphi }-1)+1$$

where, $$\chi =\frac{1+{{\rm{\Psi }}}_{Sb}/{K}_{Sb}}{1+{{\rm{\Psi }}}_{As}/{K}_{As}}$$.

Here, x and ϕ are Sb compositions in the NWs and in the incident flux (influx) on the droplets, respectively, while K_As(Sb)_ and Ψ_*As*(*Sb*)_ are the coefficients determining the liquid-to-solid incorporation rate of Ga-As(Sb) pairs and desorption of As(Sb) from the droplet. As evident from the definition of the parameter *χ*, an increase in the liquid-to-solid incorporation rate of Ga-Sb pairs and in the desorption coefficient of Sb from the droplet will respectively increase and decrease the Sb composition in the NWs. However, a similar variation in the As parameters will have an opposite impact on the Sb composition in the NWs. In general, both K_As/Sb_ and Ψ_As/Sb_ will increase with substrate temperature. Thus, a relative change in these parameters will determine the Sb composition in the NWs for a given influx composition. Due to the lower volatility of Sb as compared to As, the decrease in Ψ_*Sb*_ will be relatively higher than in Ψ_*As*_ with a decrease in temperature, and the liquid-solid phase diagram of the GaAs-GaSb system also suggests that the K_Sb_/K_As_ ratio will be higher^40^ at lower growth temperatures. Consequently, the Sb composition in the NWs will be enhanced when grown at lower substrate temperatures, which is consistent with our experimental observations.

### Two-step growth process

**Figure 4:**
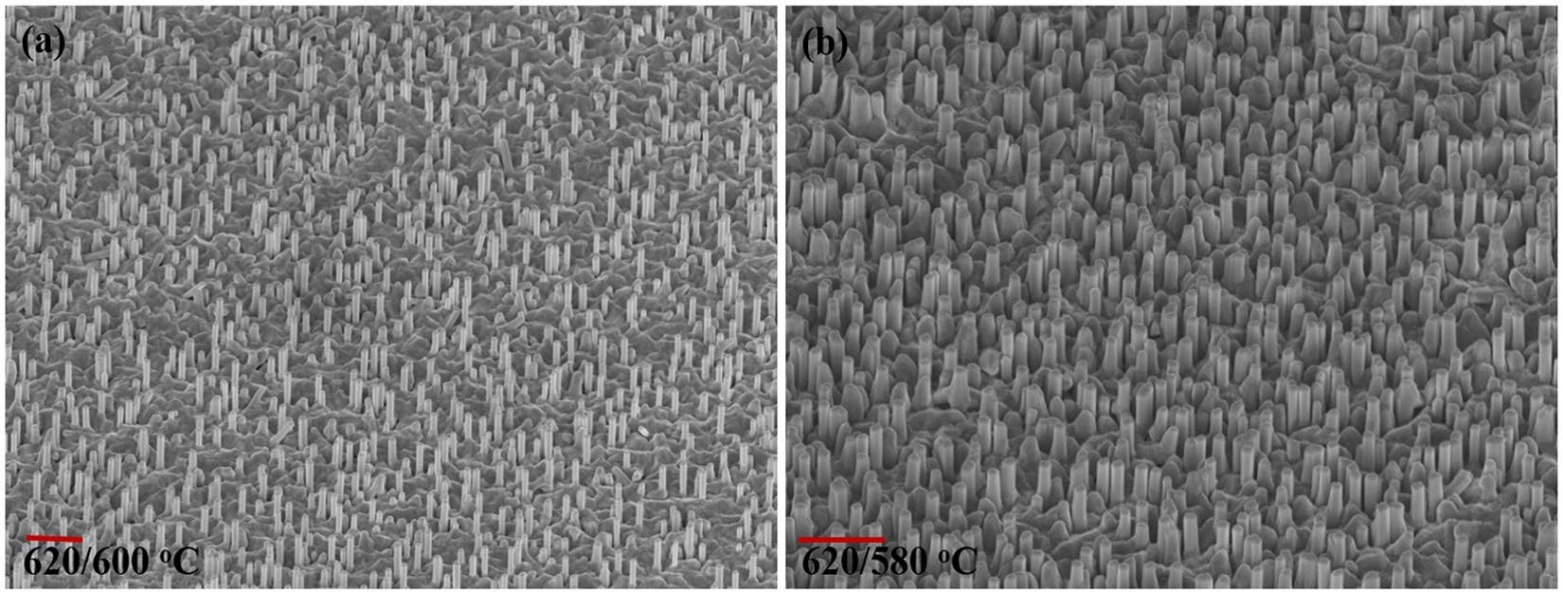
Two-step growth process: After two minutes of growth, the substrate temperature was reduced to (**a**) 600 °C and (**b**) 580 °C (the scale bar represents 1 µm).

To further take advantage of the increased Sb incorporation in the NW at lower growth temperatures without adversely affecting the NW density, a two-step growth process was examined. In this process, growth was initiated at a higher substrate temperature of 620 °C for two minutes, followed by subsequent reduction in the substrate temperature to the desired temperature. This led to significant improvement in the NW density as displayed in Fig. 4.

**Figure 5:**
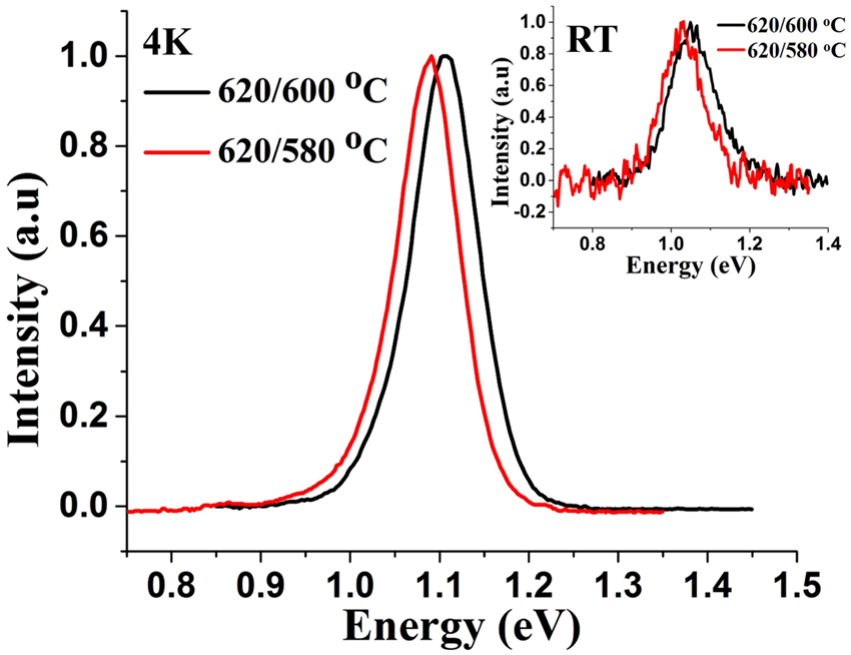
Normalized PL spectra of GaAsSb NWs with 24 at.% Sb and 25 at.% Sb compositions. The inset shows the room-temperature PL spectra of the NWs. NWs were grown by the two-step 620 °C/600 °C and 620 °C/580 °C process.

The sample with reductions in temperature from 620 °C to 600 °C and 580 °C yielded NWs of good density (~4 × 10^8^ cm^−2^) and an improvement in length of the NWs to 1 µm and 0.75 µm, respectively. Although the substrate temperature reduction was doubled in the latter sample, no significant change in Sb incorporation was observed. The Sb composition of the samples was ~24 at.% with 4 K and room- temperature PL peak energy occurring at 1.1 eV and 1.05 eV, respectively (see Fig. 5).

### Two-step Growth with Variation in the Ga flux

In the two-step growth process, the axial growth rate was significantly reduced approximately threefold despite the increase in NW density. Hence, growth was performed by doubling the growth duration at the lower growth temperature of 590 °C.

**Figure 6:**
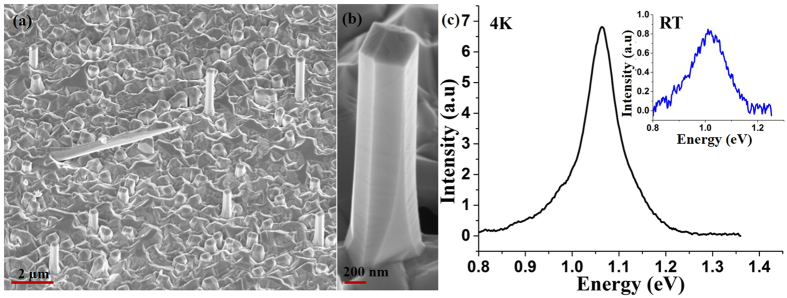
SEM images of NWs grown by the two-step growth processes for the growth temperatures of (**a**), (**b**) 620 °C/590 °C ensemble and single NW, respectively and the (**c**) 4 K PL spectra of NWs with 29 at.% Sb composition. The inset shows the room-temperature PL spectra of the NWs.

However, as demonstrated by Fig. 6a, this did not lead to a proportional increase in length, which was limited to 0.7 µm with a small percentage of the NWs exhibiting lengths in the range of 1.5–2 µm. The overall density (including the short NWs) was ~1.5 × 10^8^ cm^−2^. However, the nanowire diameter was significantly enhanced to 600 nm. The Sb composition in the NW was measured to be ~29 at.%. The 4 K and room-temperature PL spectra peaks occurred at 1.07 eV and 1.02 eV, respectively (see Fig. 6c).

The invariance of the NW length with total growth time suggests cessation of the VLS growth mechanism at some point prior to shutting of the molecular beam flux of the growth species. We ascribe this cessation to total consumption of the Ga droplet at the top of the NW. A smaller surface energy and a larger atomic number of Sb as compared to As favors the segregation of Sb, resulting in the formation of a floating layer of Sb on the surface of the Ga droplet which obstructs the collection of growth species by the droplet^41^. Decrease in the desorption rate of Sb from the droplet surface at lower temperature due to its lower volatility further impedes the replenishment of the Ga droplet, resulting in its gradual consumption and leading to termination of VLS growth. Therefore, after a certain period of time the axial growth of NWs ceases, and the supplied fluxes contribute only to radial growth, which is corroborated by the similar lengths but much larger diameters for NWs grown for a longer duration as compared to those for a shorter duration. However, the time for total consumption of the droplet will largely depend on its initial volume, BEP of Ga flux and diffusion length of Ga. The larger initial volume of some droplets is likely to have resulted in longer NWs than the average in the present growth. It is to be noted that radial growth which is enhanced by the presence of Sb in the growth environment follows the vapor-solid (VS) mechanism^23^.

**Figure 7:**
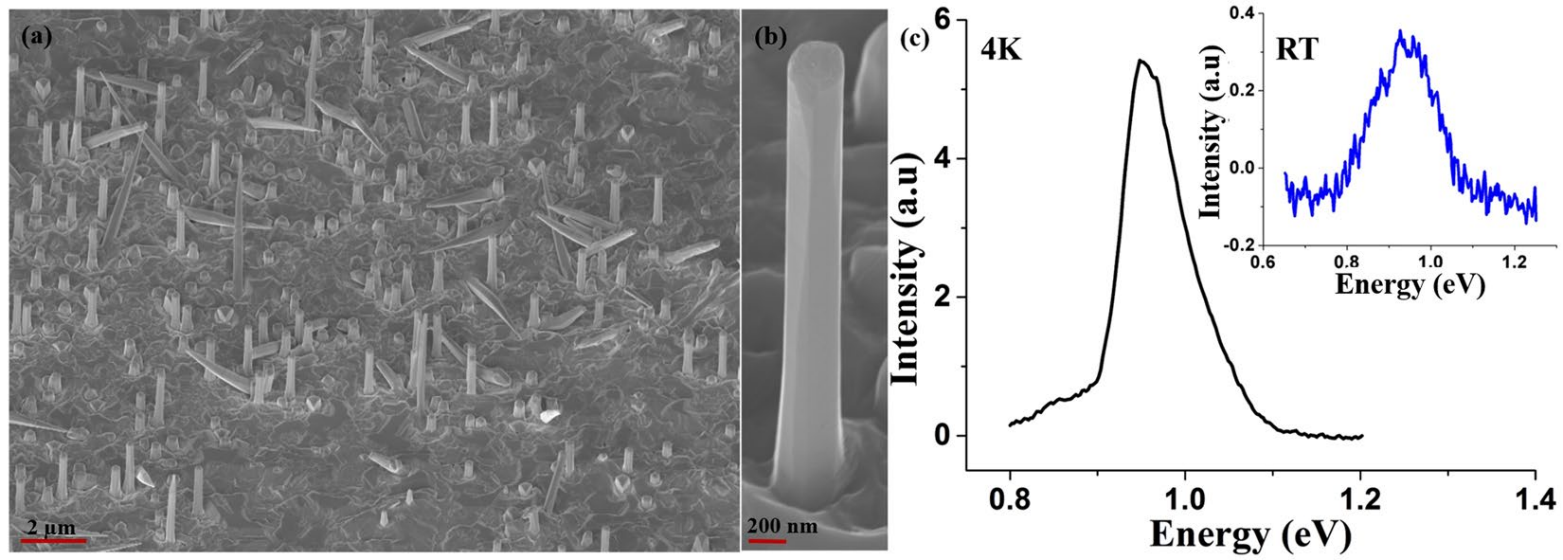
SEM images of NWs grown by two-step growth processes with Ga flux variation for the growth temperatures of (**a**), (**b**) 620 °C/590 °C ensemble and single NW, respectively and (**c**) 4 K PL spectra of NWs with 34 at.% Sb composition. The inset shows the room-temperature PL spectra of the NWs.

In order to retain the Ga droplet for continual VLS growth, the BEP ratio of Ga has been gradually increased from 2.4 × 10^−7^ to 2.7 × 10^−7^ Torr, which yielded a substantial percentage of NWs exhibiting 4 µm length with diameters in the range of 400 nm (Fig. 7) and overall NW density ~2 × 10^8^ cm^−2^. EDS measurements reveal 34 at.% Sb composition in the NWs, while the 4 K and room-temperature PL peak emissions were found to be 0.95 and 0.93 eV, respectively, which corresponds to 1.3 µm emission, as shown in Fig. 7c. We also observed similar PL peak position with less intensity from a single NW at 4 K from this sample (not shown here). We attribute the increased Sb composition to the decrease in V/III ratio ensued from the increase in Ga BEP, as GaSb NWs are commonly grown under lower V/III BEP ratio than that of GaAs NWs^9^. An increase in Ga BEP will minimize Sb desorption from the NW surface since more Ga atoms are available for making bonds with Sb atoms^42^. Moreover, despite longer NWs, the absence of a Ga droplet at the tip of the NWs (Fig. 7b) indicates the termination of the VLS growth prior to the end of growth duration, leaving only the radial VS growth to continue. Less surface energy and higher mobility of Sb^12^ compared to As makes formation of the Ga-Sb pairs more likely than incorporation of Ga-As pairs on the side-facets, although the Ga-As bond is stronger than Ga-Sb bond. We suggest that all these phenomena culminate in the observed enhancement in Sb at.% in the NWs grown under these conditions.

**Figure 8:**
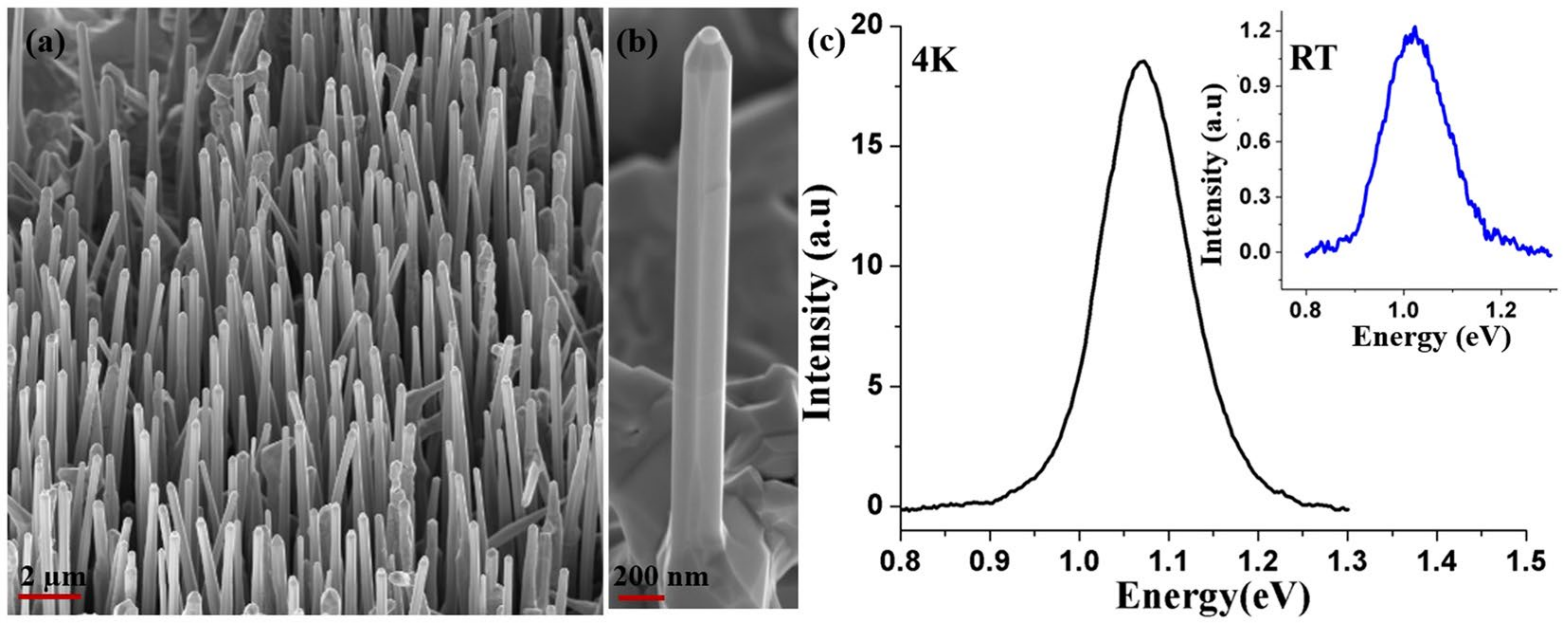
(**a**) and (**b**) show the SEM images of GaAsSb ensemble and single NW with two-step growth process of 620 °C/590 °C respectively, where Ga flux was varied during the growth. (**c**) 4 K PL spectra of NWs with the room-temperature PL spectra in the inset.

In order to further improve the NW density and uniformity in length, the Ga flux was further gradually increased from 2.4 × 10^−7^ to 3.2 × 10^−7^ Torr during growth with all other growth parameters held constant (see Fig. 8). As shown in Fig. 8a, NW density improved to 3 × 10^8^ cm^−2^ with the average length and diameter of the NWs being 5 µm and 340 nm, respectively. The Sb composition in the NW was ~28 at.% with 4 K and room-temperature PL emission at 1.08 eV and 1.03 eV, respectively. Close observation of Fig. 8b reveals the presence of a Ga droplet at the tip of the NW, which is indicative of the competition between axial and radial growth throughout the entire growth period, which explains the reduction in Sb composition. Thus, NWs of high spatial density, uniform length and smooth morphology were finally achieved by continual tuning of the V/III BEP ratio and substrate temperature during the growth.

**Figure 9:**
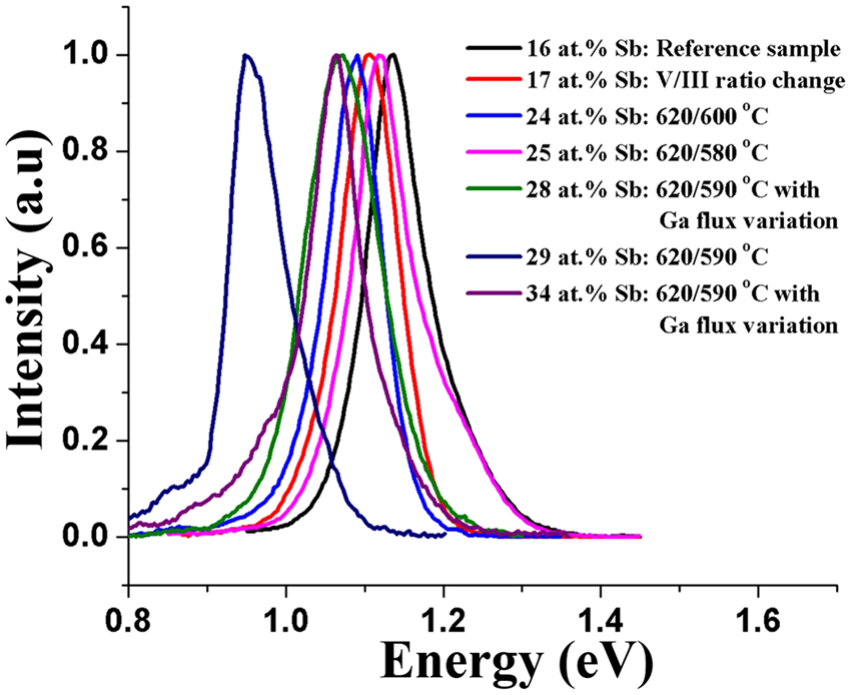
Low-temperature (4 K) normalized PL spectra of GaAsSb NWs reaching 1.3 µm emission by increasing Sb composition in the NWs using different growth parameters.

Figure 9 summarizes the above data on bandgap tuning of GaAsSb NWs with increasing Sb incorporation leading to 1.3 μm 4 K PL emission by different growth procedures. On the basis of the data, it is clear that adoption of a two-step growth sequence with Ga flux variation enables one to achieve higher Sb compositions and as a consequence longer wavelength emission.

**Figure 10:**
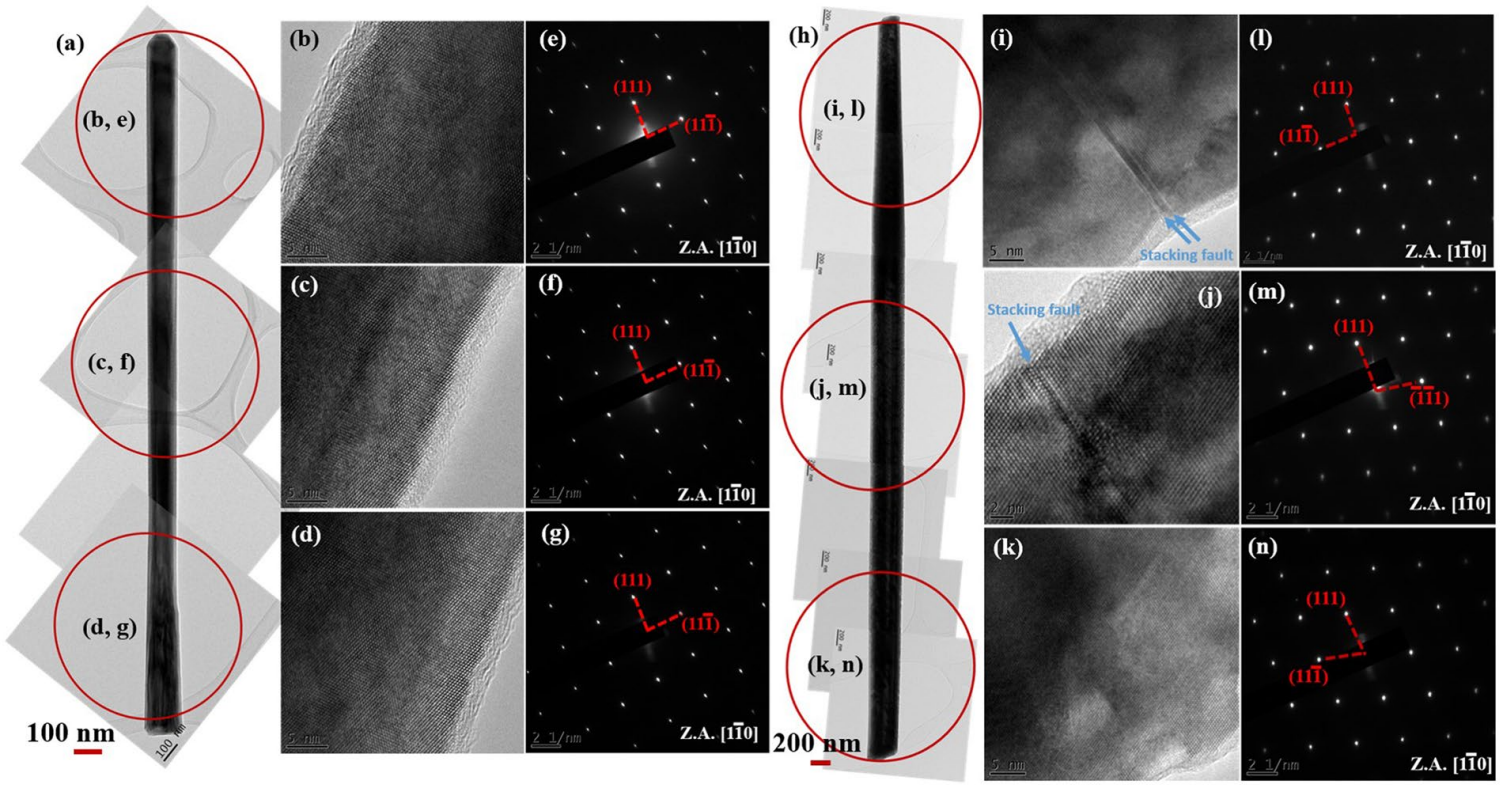
(**a**), (**h**) TEM images of GaAsSb NWs with Sb compositions of 17 at.% and 28 at.% grown by varying the V/III ratio to 18 and using two step growth process with Ga flux variation, respectively. The (**b**–**d**), (**i**–**k**) HRTEM images and (**e**–**g**), (**l**–**n**) SAED patterns are taken from top, middle and bottom locations of the respective NWs.

Figure 10a,b to d and e to g display the TEM, HRTEM and selected area electron diffraction (SAED) patterns of GaAsSb NW with an Sb composition of 17 at.%, grown for a V/III ratio of 18. These HRTEM images and corresponding SAED patterns acquired from three different positions along the NW confirm pure zinc blende (ZB) crystal structure without noticeable planar defects. Lack of any twins in the NWs is also supported by the absence of any satellite diffraction spots in the SAED patterns. Figure 10h,i to k and l to m shows the TEM, HRTEM and SAED patterns of GaAsSb NW with Sb composition of 28 at.%, grown by our novel two step growth process with variation in the Ga flux during the growth. HRTEM images at the top and middle locations identified with blue arrows are indicative of presence of stacking faults.

**Figure 11:**
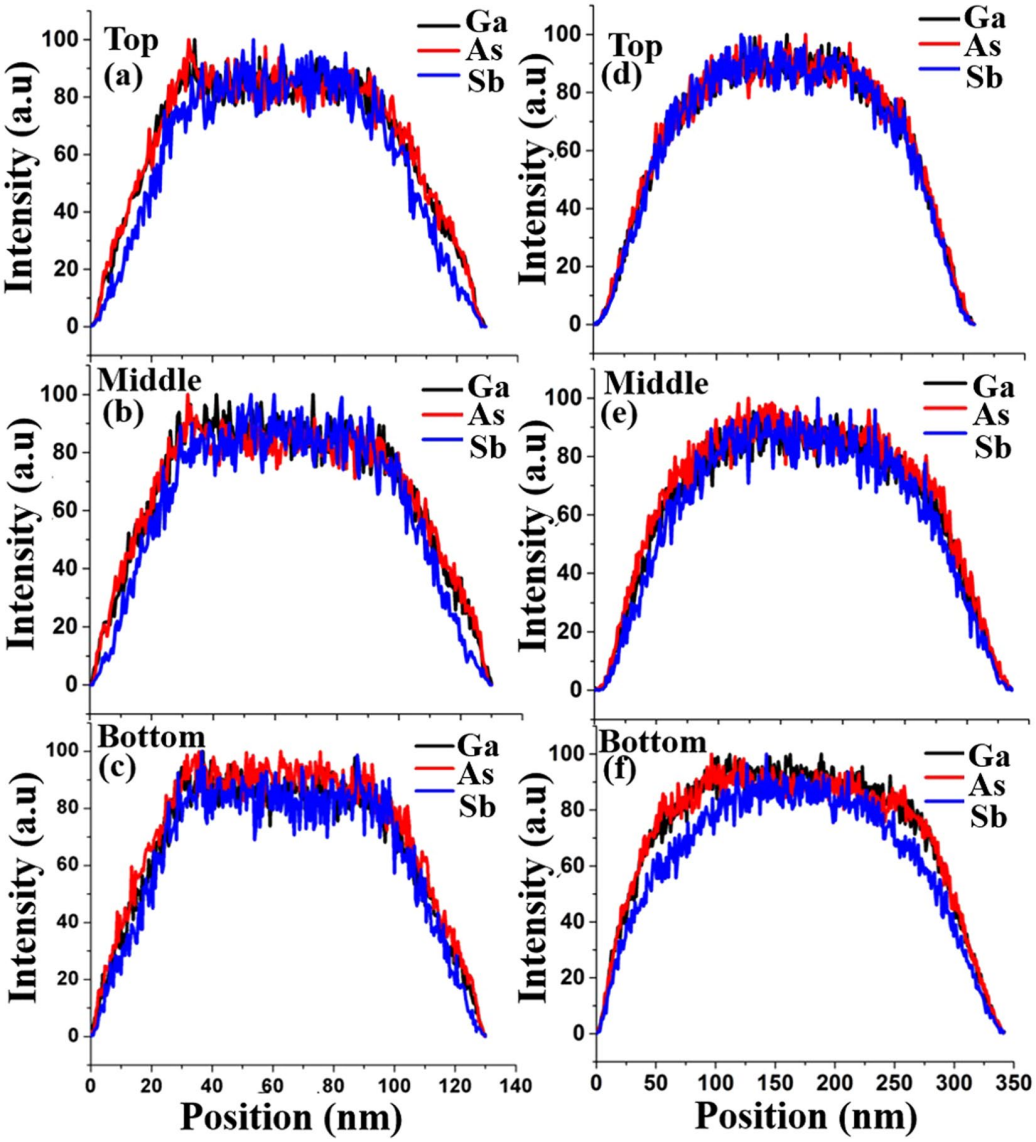
(**a**–**c**) and (**d**–**e**) show EDS line scans at top, middle and bottom segments for 17 at.% and 28 at.% Sb contents GaAsSb NWs grown for V/III ratio of 18 and two step growth with variation in Ga flux during the growth, respectively.

Figure 11a to c exhibits EDS line scans at top, middle and bottom segments of the GaAsSb NWs with Sb composition of 17 at.% grown for V/III ratio of 18. Good compositional homogeneity with a plateau of about 70 nm at the center of the core of the NWs was observed with gradual termination of all the constituents within an outer boundary of ~20 nm on either side of the NWs. However, the NWs with an Sb composition of 28 at.% using the two-step growth process with varying Ga flux during the growth exhibited gradual tapering of the elemental constituents with an outer boundary extending to ~100 nm from the center of the core to the edges, as revealed in the EDS line scan of the three segments of the NWs in Fig. 11d to f. The variation in the behavior can be attributed to the differences in the growth mechanisms. Higher radial growth rate due to higher Sb incorporation at reduced substrate temperature for the case of NW of 28 at.% (Fig. 11d to f) in comparison to the NWs of 17 at.% Sb composition (Fig. (a to c)) suggests that a larger fraction of cross section of the former NW has been formed by the VS mechanism, resulting in more rounded surface morphology with gradual decline in the constituent elements at the edges.

### XRD and Raman Analysis

**Figure 12:**
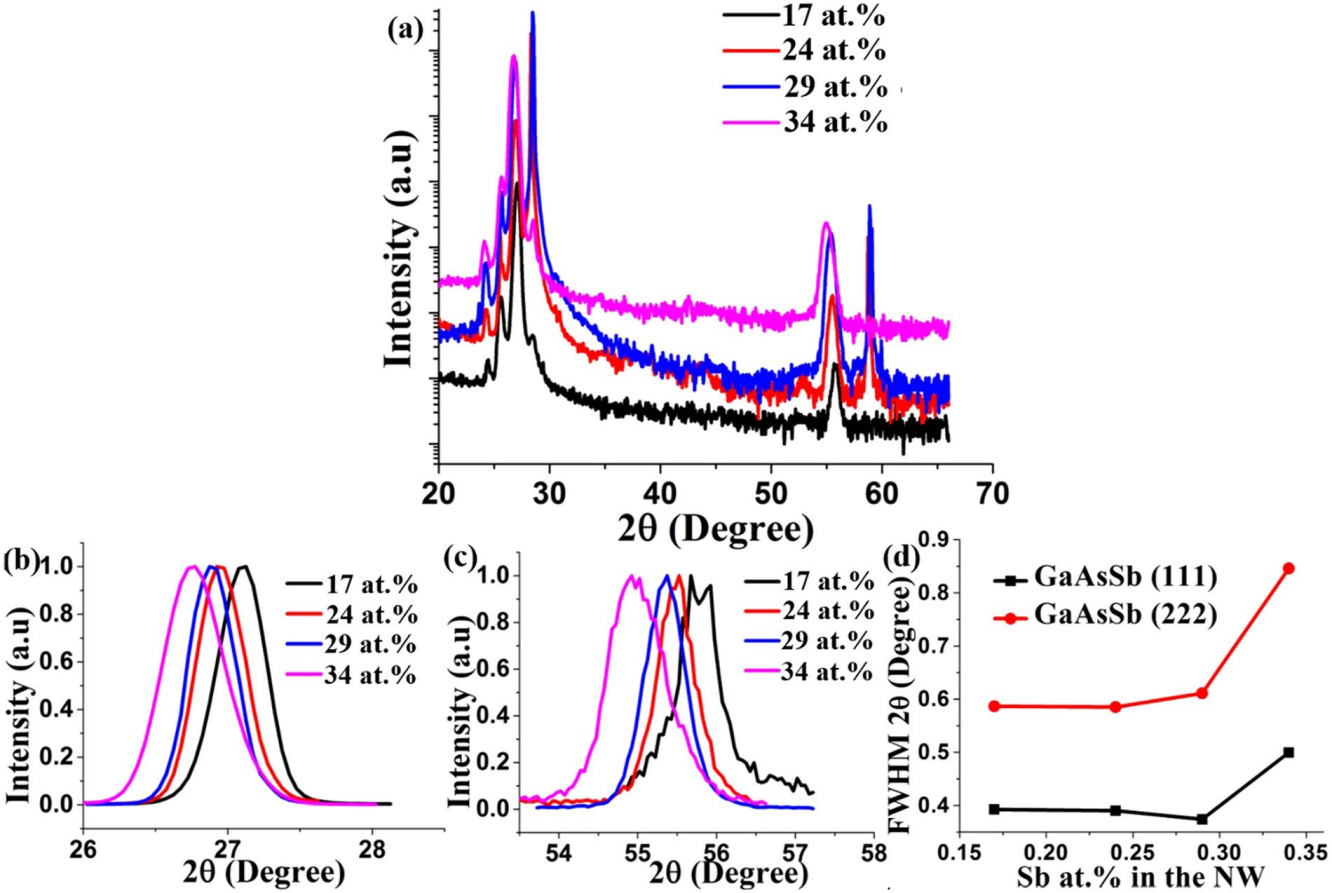
XRD spectra of GaAs_1−x_Sb_x_ NWs for (**a**) different Sb compositions; (**b**) and (**c**) represent the corresponding GaAsSb (111) and GaAsSb (222) Bragg peaks; (**d**) represent the change in FWHM with Sb at.% variation in the NWs.

Figure 12 shows the X-ray diffraction spectra of GaAs_1−x_Sb_x_ NWs for different Sb compositions. All the diffraction peaks were identified from the Joint Committee on Powder Diffraction Standards (JSPDS) database. The presence of only (111) oriented GaAsSb and Si Bragg peaks and their higher order reflections are clear evidence of well-aligned (111) oriented NWs. In the case of 17 at.% and 34 at.% Sb, the Si Bragg peaks are weaker due to a thicker 2D layer. With increasing Sb composition in the NWs, both the GaAsSb (111) and GaAsSb (222) Bragg peaks exhibit shifts toward a lower angle (Fig. 12b,c) accompanied with a larger FWHM (Fig. 12d) of the spectra for 34 at.% Sb, attesting to the increased incorporation of larger atomic size Sb in the lattice that leads to enhanced disorder-induced strain^22, 33^.

**Figure 13:**
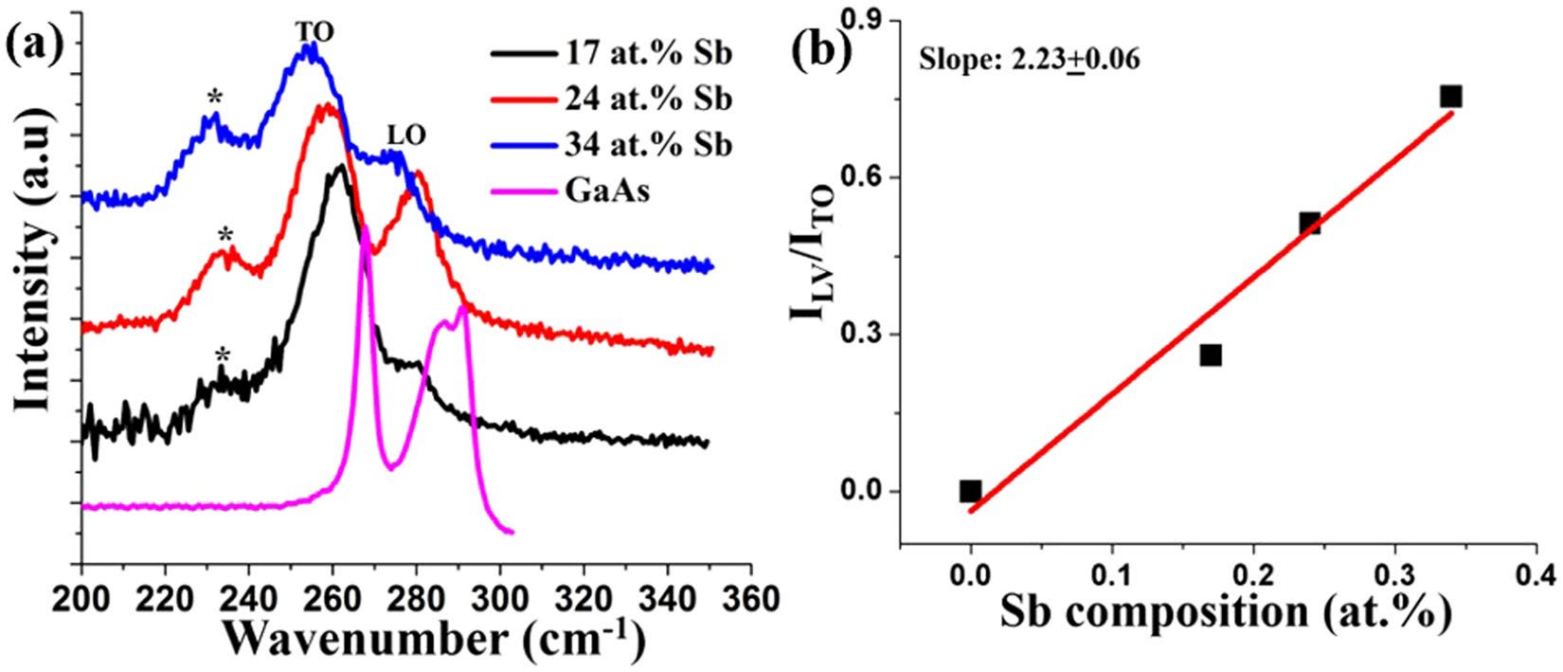
(**a**) Room-temperature Raman spectra of GaAsSb NWs at different Sb compositions, the spectra are vertically shifted for better clarity. (**b**) Normalized GaSb related LV mode as a function of Sb composition in the NWs, red line indicates the linear fit to the experimental data.

Figure 13a shows the room-temperature Raman spectra of GaAs_1−x_Sb_x_ NWs at different Sb compositions along with the Raman spectra of the reference GaAs NWs. GaAs NWs exhibit sharp TO and LO modes at 267.8 cm^−1^ and 291 cm^−1^, respectively. The Raman spectra of GaAsSb NWs are quite distinct from that of the GaAs NWs. In the latter, both the peaks are sharp with the LO /TO peak ratio being higher with a shoulder at 287 cm^−1^ present on the higher wavenumber LO mode, which is attributed to a surface optical mode (SO)^10, 20^ that is commonly observed in thin NWs. With increasing Sb content to 34 at.%, both TO and LO mode signals are broadened with a maximum red shifts to 254 cm^−1^ and 275 cm^−1^, respectively. This red shift of the phonon modes is influenced by different material characteristics, namely mass disorder, change in the dielectric medium and strength of the ionic plasma coupling associated with alloying^20, 22^, and both LO and TO modes increase with higher Sb composition in the NWs. The broadening of the modes with Sb composition is indicative of a higher degree of disorder. The nearly symmetric shape of the phonon modes for axial GaAsSb NWs, unlike those observed in core-shell GaAsSb NWs^10^, is indicative of the absence of any noticeable planar defects in the NWs, which is consistent with our earlier TEM data.

Another interesting feature that is observed in Fig. 13a is an increase in the LO/TO ratio with increasing Sb composition, which we attribute to the increase in NW diameter. A strong dependence of LO and TO mode intensities on nano-crystallite size (R) has been reported^43^. The phonon mode contributions to the decay rate of the phase relaxation of the excited states of the phonons impacting the LO/TO intensity ratio is predicted to vary as 1/R^2.5^, which explains the observed higher LO/TO ratio with Sb composition in NWs of larger diameter.

An additional peak near 230 cm^−1^ was also observed, as denoted by the asterisk in Fig. 13a. The origin of this peak is associated with the local vibrational mode (LV) of GaSb in the GaAs lattice^20^ and is commonly observed in III-V ternary alloy systems^44–46^, which becomes stronger with increasing Sb at.%. The relative intensity of the GaSb LV mode depends on the Sb concentration in the GaAsSb NWs, as demonstrated in Fig. 13b in which the relative intensity of the ratio of the LV mode to the normalized TO mode has been plotted as a function of Sb concentration in the NWs. A linear fit resulted in a slope of 2.23, which is comparatively higher than the slope of ~1.2 reported in the literature^20, 22^. The explanation for the observed larger slope in our work is as follows. It has been reported that cross-section EDX mapping of GaAsSb NWs when grown by the traditional VLS procedure exhibits ~10% less Sb at the apexes and interfaces of the <112> corners of the NWs, which results in As-rich bands inside the NWs due to the high diffusion length of Sb^19, 47^. In the two-step growth procedure, the diffusion length of Sb is expected to be lower, as the GaAsSb growth is carried out at lower growth temperatures. In our case, the comparatively stronger GaSb LV mode at similar Sb composition indicates that those As-rich bands weaken due to the two-step growth process, which allows formation of more GaSb bonds in the NWs. Presence of this excess in GaSb bonds and weaker As-rich bands are the most likely cause for our observation of a strong GaSb LV mode and hence higher slope in our case although further investigation is required to confirm this hypothesis.

Thus, we have demonstrated a potential pathway to achieve the telecommunication wavelength of 1.3 µm in GaAsSb NWs in an axial configuration. These nanowires exhibit superior optoelectronic properties as compared to those of a core-shell configuration since the latter has been reported to suffer from a high density of planar defects, strain-induced bending and multiple PL peaks due to the presence of type-I and type-II transitions. The two-step growth technique can also further be extended to site-selective patterned NWs, thus making it a viable technique for future large-scale commercialization of GaAsSb nanowire-based optoelectronic devices.

## Conclusions

From the comprehensive and systematic study of the MBE growth of GaAsSb NWs in the axial configuration as a function of constituent growth flux and substrate temperature, we have demonstrated that the challenge of rapid consumption of the Ga melt at the tip by the excess group V flux at higher Sb concentration can be overcome by a two-step growth process with gradual variation of the Ga supply during axial growth. Using this process, we have demonstrated the growth of GaAsSb nanowires in an axial configuration that exhibit PL emission at 1.3 μm, which reveals a promising pathway for bandgap tuning NWs. The narrow FWHM of the PL peak and the lack of any noticeable twins in the corresponding HRTEM images and SAED diffraction pattern are indicative of high quality microstructures. The insight provided by our study on the influence of V/III ratio, growth temperature and thermodynamic relation between vapor flux and NW composition modulation may also be generally applicable to other ternary III-V NW systems.

### Data Availability

All data generated or analyzed during this study are included in this published article (and its supplementary information file).

## Electronic supplementary material

Supplementary information
