# Supplementary material for: A Two-Step Growth Pathway for High Sb Incorporation in GaAsSb Nanowires in the Telecommunication Wavelength Range
Source: Sci Rep. 2017 Aug 31;7:10111. doi: 10.1038/s41598-017-09280-4 (PMC5579295; doi:10.1038/s41598-017-09280-4)
Supplement: Supplementary file 1 — Supplementary information [file 41598_2017_9280_MOESM1_ESM.pdf]

A Two-Step Growth Pathway for High Sb Incorporation in GaAsSb Nanowires in the Telecommunication Wavelength Range

Estiak Ahmad, Md Rezaul Karim, Shihab Bin Hafiz, C Lew Reynolds Jr., Yang Liu and *Shanthi Iyer

1. Theoretical Model for Nanowire Composition

Let C_{Ga}, C_{As} and C_{Sb} be the concentrations of Ga, As and Sb, respectively, in the droplet during the self-catalyzed VLS growth of GaAsSb NWs of axial configuration where $C_{Ga}+C_{As}+C_{Sb} = 1$. Considering the fact that, axial growth rate is limited by the availability of the group-V species[23,49], the steady state balance equations of transformation of the unbound Ga-As and Ga-Sb pairs in the liquid to the corresponding bound pair in solid can be expressed as

$$K_{As}C_{As} = \Phi_{As} - \Psi_{As}C_{As} \quad \text{(Eq. S1)}$$
$$K_{Sb}C_{Sb} = \Phi_{Sb} - \Psi_{Sb}C_{Sb} \quad \text{(Eq. S2)}$$

Here, the parameters K, Φ and Ψ determine the liquid to solid incorporation rates, influx to and efflux from the Ga droplet of the subscripted growth species. It should be noted that, these equations are similar to those described by Dubrovskii in Ref. 50 under the condition $C_{Ga} = 1$ which is a reasonable assumption for the case of self-catalyzed growth. Adopting the definitions of Dubrovskii[50] composition of Sb in the NW, droplet and influx can be expressed, respectively, as

$$x = \frac{K_{Sb}C_{Sb}}{K_{As}C_{As} + K_{Sb}C_{Sb}}$$
$$y = \frac{C_{Sb}}{C_{As} + C_{Sb}}$$

and

$$\phi = \frac{\Phi_{Sb}}{\Phi_{As} + \Phi_{Sb}}$$

It should be noted that, influx composition or in other words effective vapor composition, ϕ is expected to be different from those actually present in the primary molecular beam flux, due to the difference in - the diffusivity of group-V adatoms on the substrate, the solubility of As and Sb in the Ga droplet[36] and the reemission probability of As and Sb from substrate surface and NW side-facets[23]. Using the relations in Eqs. S1 and S2, NW composition, x and composition, ϕ can be related as

$$\frac{\mathbf{1}}{\boldsymbol{x}} = \chi\left(\frac{\mathbf{1}}{\boldsymbol{\phi}} - \mathbf{1}\right) + 1 \quad \text{(Eq. S3)}$$

where, $\chi = \frac{1+\Psi_{Sb}/K_{Sb}}{1+\Psi_{As}/K_{As}}$.

The Eq. S3 suggests a positive correlation between the Sb compositions in the influx and NW. Moreover, the expression of the parameter χ is indicative of possible approaches for tuning the NW composition while keeping the effective vapor composition unaltered. Intuitively, an increase in the liquid-to-solid incorporation rate of Sb (As) should increase (decrease) the Sb composition in the NW, consistent with Eq. S3. On the other hand, efflux coefficients Ψ_{Sb} and Ψ_{As} affect the NW composition in exactly opposite manner. All of these four parameters can be altered by

changing the growth temperature; in fact, increase in temperature will increase both the incorporation rates and efflux coefficients and their relative change will be the determinate factor of the variation in NW composition. For example, an increase in substrate temperature will increase both the Ψ_{Sb} and Ψ_{As} and due to the higher vapor pressure of Sb relative change in Ψ_{Sb} will be greater than that in Ψ_{As} , ensuing a decrease in Sb composition in the NW. Finally, Eq. S3 suggests that unlike the Au-catalyzed growth of NWs, the effect of V/III ratio in the vapor will have minimal effect in determining the NW composition. However, the effect of V/III ratio on the chemical potential of group-V species in the droplet will be observed in the changes in the nucleation probability as well as the NW growth rate.

2. Comparison of PL peak position with Sb variation in GaAsSb:

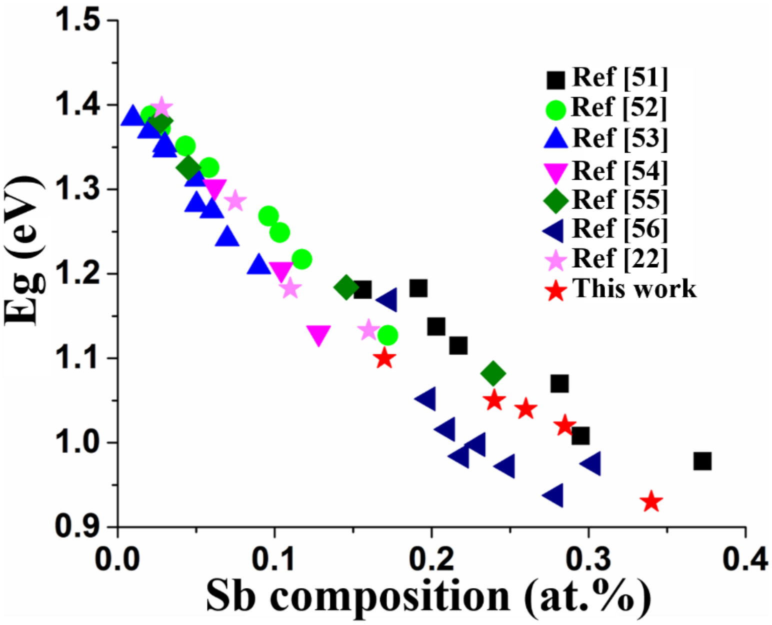

Figure S1: Comparison of PL peak position variation of GaAsSb NWs at room temperature along with the bandgap energies (E_g) reported in quantum wells and thin films as a function of Sb concentration.

In Fig. S1 a comparison of PL peak energy variation with Sb composition from this work with those reported in literature on bandgap energy of GaAsSb quantum well (QW), thin films and NWs have been shown, which reveal an excellent agreement.

References

49. Gibson, S. J. & LaPierre, R. R. Model of patterned self-assisted nanowire growth Nanotechnology 24, 415304 (2014).

50. Dubrovskii, V. G. Fully analytical description for the composition of ternary vapor−liquid−solid nanowires *Cryst. Growth Des.* 15, 5738−5743 (2015).

51. R. Teissier, D. Scicault, J. C. Harmand, G. Ungaro, G. L. Roux, L. Largeau 2001 J. Appl. Phys. 89 10 5473

52. R. E Nahory, M. A. Pollack, J. C. DeWinter and K. M. Williams 1977 J. Appl. Phys. 48 1607

53. J. L. Castano and J. Piqueras 1983 J. Appl. Phys. 54 3433

54. M. B. Thomas, W. M. Coderre and J. C. Woolley 1977 Phys. Status Solidi A 2 K141

55. G. A. Antypas and L. W. James 1970 J. Appl. Phys 41 2165

56. R. M. Cohen, M. J. Cherng, R. E. Benner and G. B. Stringfellow 1985 J. Appl. Phys 57 4817
